# Preserved C-peptide is common and associated with higher time in range in Chinese type 1 diabetes

**DOI:** 10.3389/fendo.2024.1335913

**Published:** 2024-02-09

**Authors:** Wei Liu, Yayu Fang, Xiaoling Cai, Yu Zhu, Mingxia Zhang, Xueyao Han, Juan Li, Sai Yin, Deheng Cai, Jing Chen, Lei Wang, Dawei Shi, Linong Ji

**Affiliations:** ^1^ Department of Endocrinology and Metabolism, Peking University People’s Hospital, Beijing, ;China; ^2^ School of Automation, Beijing Institute of Technology, Beijing, ;China

**Keywords:** type 1 diabetes, preserved C-peptide, beta cell, CGM, glycemic control

## Abstract

**Objective:**

The aim of this study is to determine the residual C-peptide level and to explore the clinical significance of preserved C-peptide secretion in glycemic control in Chinese individuals with type 1 diabetes (T1D).

**Research design and methods:**

A total of 534 participants with T1D were enrolled and divided into two groups, low–C-peptide group (fasting C-peptide ≤10 pmol/L) and preserved–C-peptide group (fasting C-peptide >10 pmol/L), and clinical factors were compared between the two groups. In 174 participants who were followed, factors associated with C-peptide loss were also identified by Cox regression. In addition, glucose metrics derived from intermittently scanned continuous glucose monitoring were compared between individuals with low C-peptide and those with preserved C-peptide in 178 participants.

**Results:**

The lack of preserved C-peptide was associated with longer diabetes duration, glutamic acid decarboxylase autoantibody, and higher daily insulin doses, after adjustment {OR, 1.10 [interquartile range (IQR), 1.06–1.14]; OR, 0.46 (IQR, 0.27–0.77); OR, 1.04 (IQR, 1.02–1.06)}. In the longitudinal analysis, the percentages of individuals with preserved C-peptide were 71.4%, 56.8%, 71.7%, 62.5%, and 22.2% over 5 years of follow-up. Preserved C-peptide was also associated with higher time in range after adjustment of diabetes duration [62.4 (IQR, 47.3–76.6) vs. 50.3 (IQR, 36.2–63.0) %, adjusted *P* = 0.003].

**Conclusions:**

Our results indicate that a high proportion of Chinese patients with T1D had preserved C-peptide secretion. Meanwhile, residual C-peptide was associated with favorable glycemic control, suggesting the importance of research on adjunctive therapy to maintain β-cell function in T1D.

## Introduction

Type 1 diabetes (T1D) is characterized by progressive autoimmune destruction of β cells. The loss of β cells leading to the diagnosis of T1D is gradual and continues after clinical onset. Initially, a significant number of β cells remain, and relatively low doses of exogenous insulin are required to limit glucose variability and hypoglycemia. Although it has been assumed that β cells are irreversibly lost after diagnosis, recent studies have shown that not all β cells are destroyed and that many people with T1D continue to produce insulin even after long-term disease course (1, 2). The Diabetes Control and Complications Trial showed that the persistence of residual β cells, as measured by C-peptide secretion, is associated with better glycemic control, reduced glycemic variability, and a lower incidence of microvascular complications (3, 4). Understanding the presence and trends of residual β-cell function and its relationship to the heterogeneity of glycemic control may provide insights into the natural history of the disease and facilitate possible interventions to modify disease progression.

Previous studies have suggested heterogeneity in preserved β-cell function in T1D across cohorts and according to the definition of “preserved C-peptide secretion.” In the Scottish Diabetes Research Network Type 1 Bioresource cohort, 37.7% of participants retain detectable non-fasting C-peptide (>5 pmol/L) (5). In addition, in the T1D Exchange Clinic Network, detectable non-fasting C-peptide (>17 pmol/L) was found in 29% of participants, and the frequency of non-fasting C-peptide ≥200 pmol/L was 10% (6). Meanwhile, even minimal levels of C-peptide have clinical significance in established T1D. Kuhtreiber et al. found that fasting C-peptide levels >10 pmol/L were associated with protection from complications (7), and Fraser et al. found that, under the same definition of preserved C-peptide, it was associated with fewer low glucose events and lower glucose variability on intermittently scanned continuous glucose monitoring (isCGM) (8). Although the maintenance of C-peptide secretion has been well studied in the Caucasian population, little is known about non-Caucasian populations, particularly East Asians. The aim of this study was to evaluate residual β-cell function, the underlying clinical factors contributing to the preservation of C-peptide secretion, and its impact on glycemic control in Chinese individuals with T1D.

## Research design and methods

### Study design and participants

**Figure 1:**
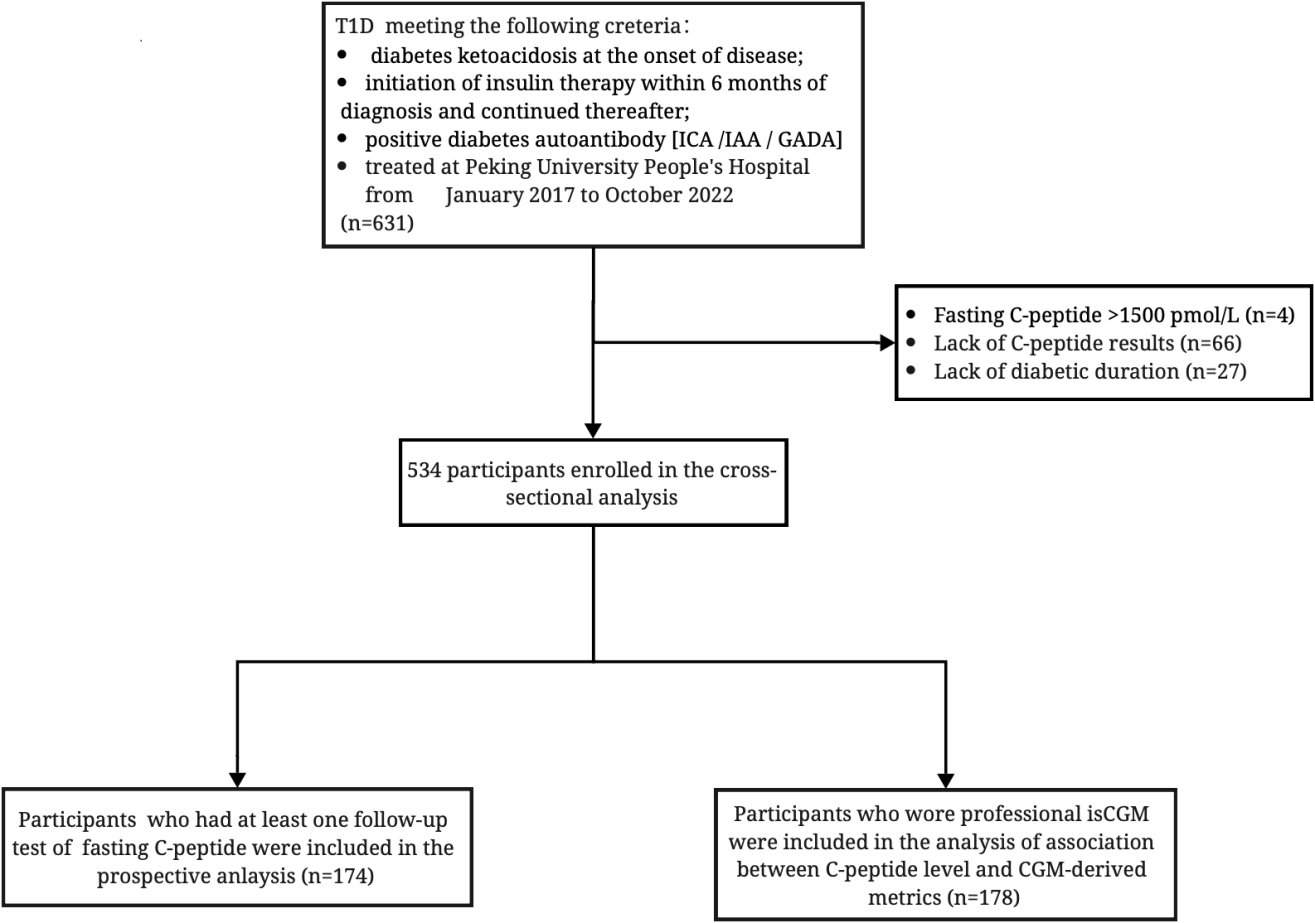
Inclusion flowchart of 534 participants with diabetic duration information and fasting C-peptide data; 174 participants had a at least one follow-up test of fasting C-peptide test; 178 participants had CGM-derived data. ICA, islet cell autoantibody; IAA, insulin autoantibody; GADA, glutamic acid decarboxylase autoantibody.

A total of 631 individuals with T1D treated at Peking University People’s Hospital from January 2017 to October 2022 were screened for eligibility. The diagnosis of T1D was made independently by two endocrinologists based on clinical manifestations: diabetes ketoacidosis at the onset of disease, initiation of insulin therapy within 6 months of diagnosis and continued thereafter, or positive diabetes autoantibody [islet cell autoantibody (ICA)/insulin autoantibody (IAA)/glutamic acid decarboxylase (GAD) autoantibody]. Moreover, individuals with fasting C-peptide >1,500 pmol/L were excluded to limit the possibility of including people with diagnoses other than T1D (N = 4). Sixty-six participants lacking the data of C-peptide and 27 participants lacking the information of diabetes duration were excluded. Cross-sectional analysis were performed in the remained 534 participants. Of the participants, 174 people who returned to the clinic and had regular β-cell function assessments were included in the longitudinal analysis to determine the change in C-peptide secretion over the course of the disease. Meanwhile, 178 participants who wore professional isCGM were also included for analysis of glucose control according to C-peptide levels (**Figure 1**).

The study was conducted in accordance with the ethical principles in the Declaration of Helsinki and was approved by the Peking University People’s Hospital Ethics Committee (2022PHB407-001). Informed consent was obtained from all participants.

### Physical and laboratory measurements

Blood samples were taken in the morning after an 8-h to 10-h fast, and a mixed meal tolerance test (MMTT) was performed (9). During the MMTT, participants consumed a standardized breakfast calculated on the basis of total caloric requirements (25%–30% of daily caloric intake; 50% of calories as carbohydrates, 33% of calories as lipids, and 17% of calories as proteins). Glucose, low-density lipoprotein cholesterol (LDL-C), triglycerides, and uric acid were measured using an automated biochemistry analyzer. HbA_1c_ was measured by high-performance liquid chromatography (Primus Ultra 2, Trinity Biotech, Bray, Co-Wicklow, Ireland). Insulin and C-peptide were assayed by electrochemiluminescence immunoassay on a Roche autoanalyzer (Cobas e601, Germany) using Elecsys C-Peptide (Roche Diagnostics GmbH, Mannheim, Germany). The inter-assay CVs for the low–, medium–, and high–C-peptide controls were 3.4%, 2.6%, and 1.8%, respectively.

### Professional isCGM

isCGM was placed at clinic by care givers. A professional CGM (Freestyle Libre H, Abbott, US) was used to collect glucose data every 15 min for 14 days. The glucose metrics were calculated using data from 174 participants who had sensor activation over 90% during the 14 days period. Standard deviation (SD), mean glucose (MG), coefficient of variance (CV), interquartile range (IQR), mean amplitude of glucose excursions (MAGE), time below range (TBR), time above range (TAR), and time in range (TIR) were calculated according to isCGM data.

### Statistical analyses

Unless explicitly stated otherwise, statistical analysis for this study was performed as follows. Continuous variables that followed a normal distribution were expressed as mean ± SD, whereas non-normally distributed variables were presented as median with IQR. Categorical variables were reported as proportions.

Participants were divided into a low–C-peptide group (fasting C-peptide ≤10 pmol/L) and a preserved–C-peptide group (fasting C-peptide >10 pmol/L). One-way ANOVA and Mann–Whitney U-tests were used to compare continuous variables between the two cohorts, depending on the distribution of the variables. Chi-squared tests were used for categorical variables.

Factors identified in the univariate analysis, including age at diagnosis, duration of diabetes, body mass index (BMI), positive GAD autoantibody, estimated glomerular filtration rate (eGFR), daily insulin dose, and HbA1c category were then examined using binary logistic regression analysis. In the longitudinal cohort, the change in C-peptide levels from baseline to last follow-up (ΔC-peptide _last follow-up - baseline_) was used to define individuals with sustained and failed β-cell function. Cox regression analysis was also performed to determine the influence of age at diagnosis, duration of diabetes, HbA_1c_, and positive GAD autoantibodies on β-cell function. Diabetes duration was adjusted in the logistic model to assess the association between C-peptide level and CGM metric.

Statistical analysis was performed using SPSS software (version 26), and a p-value<0.05 was considered statistically significant. R (version 4.3.2) and GraphPad Prism (version 9.3.1) were used to generate the figures.

## Results

A total of 534 people were included in the study, 46.1% of whom were men. The average age of the participants was 50 years, and the average duration of diabetes was 9 years. The average HbA_1c_ level of the participants was 8.9%, and 21.9% of the participants were under euglycemic control (HbA_1c _≤7%).

### Preserved C-peptide was common even with long duration of diabetes

Of the participants, 55.4% still had preserved C-peptide (fasting C-peptide >10 pmol/L). Among those who had diabetes for more than 20 years (n = 131), 38.9% still had detectable C-peptide levels (fasting C-peptide >3 pmol/L, **Supplementary Figure 1**). Fasting C-peptide levels decreased with diabetes duration, and the fitted curve suggested a non-linear association between C-peptide and disease duration (**Supplementary Figure 2**).

### C-peptide levels independently associated with diabetes duration and positive GAD autoantibody

Participants in the preserved–C-peptide group were younger [48 (IQR, 34–61) vs. 54 (IQR, 38–64) years, *P* = 0.002], had a shorter diabetes duration [4.0 (IQR, 0.7–12.0) vs. 15.0 (IQR, 7.0–32.0) years, *P* < 0.001], and had a lower insulin dose [29.4 (IQR, 20.0–40.0) vs. 36.0 (IQR, 28.3–46.0) U/d, *P* <0.001] compared with those in the low–C-peptide group. Meanwhile, BMI was lower in the preserved–C-peptide group than that in the low–C-peptide group (22.5 ± 3.4 vs. 23.1± 3.2 kg/m^2^, *P* = 0.027). Positive GAD autoantibody was detected in 71.9% of participants in the preserved C-peptide and 42.9% in the low–C-peptide group (*P* < 0.001). The eGFR was also higher in the preserved–C-peptide group [110.7 (IQR, 99.2–124.7) vs. 104.2 (IQR, 92.4–116.9) ml/min * 1.73 m^2^, *P*< 0.001]. In addition, the rates of diabetic retinopathy and carotid plaque were lower in the preserved–C-peptide group (24.1% vs. 39.9%, *P* = 0.003; 49.3% vs. 62.9%, *P* = 0.010) **Table 1**.

**Table 1:** Characteristics of the study participants according to the serum C-peptide level.

Characteristic	Total N = 534	Low C-peptide ≤ 10 pmol/L N = 238	Preserved C-peptide > 10 pmol/L N = 296	*P*
Age, years	50 (35, 62)	54 (38, 64)	48 (34, 61)	0.002
Male, n (%)	246 (46.1)	101 (42.4)	145 (49.0)	0.138
Smoking, n (%)	130 (30.2)	54 (28.0)	76 (31.9)	0.400
Age at diagnosis, years	36 (22, 50)	30 (17, 47)	38 (25, 52)	<0.001
Duration of diabetes, years	9.0 (2.0, 20.0)	15.0 (7.0, 32.0)	4.0 (0.7, 12.0)	<0.001
Weight, kg	59.5 (53.5, 67.6)	59.9 (54.1, 67.8)	59.3 (53.0, 67.5)	0.326
Body mass index, kg/m^2^	22.8 (3.3)	23.1 (3.2)	22.5 (3.4)	0.027
Waist-to-hip ratio	0.88 (0.83, 0.92)	0.87 (0.82, 0.92)	0.89 (0.83, 0.93)	0.084
IAA antibody positivity, n (%)	67 (14.3)	35 (16.7)	32 (12.5)	0.232
ICA antibody positivity, n (%)	13 (2.8)	7 (3.4)	6 (2.3)	0.574
GAD antibody positivity, n (%)	294 (59.2)	94 (42.9)	200 (71.9)	<0.001
HbA_1c_, n (%)				0.008
≤7%	115 (21.9)	63 (27.4)	52 (17.6)	
>7%	411 (78.1)	167 (72.6)	244 (82.4)	
SBP, mmHg	127 (116, 140)	128 (118, 140)	126 (113, 140)	0.264
DBP, mmHg	72 (66, 80)	72 (65, 80)	73 (66, 82)	0.120
TG, mmol/L	0.9 (0.7, 1.2)	0.9 (0.7, 1.2)	0.9 (0.7, 1.2)	0.951
LDL-C, mmol/L	2.5 (2.1, 3.1)	2.5 (2.1, 3.1)	2.5 (2.0, 3.1)	0.668
Urine microalbumin/creatinine ratio, mg/g	6.3 (3.1, 16.3)	7.6 (3.0, 32.3)	5.7 (3.2, 13.3)	0.065
eGFR, ml/min * 1.73m^2^	107.7 (95.9, 122.2)	104.2 (92.4, 116.9)	110.7 (99.2, 124.7)	<0.001
Fasting plasma glucose, mmol/L	9.3 (6.3, 13.4)	10.1 (6.4, 14.8)	9.0 (6.3, 12.6)	0.026
MMTT stimulated glucose, mmol/L	13.0 (5.2)	13.4 (5.7)	12.8 (4.8)	0.233
Fasting insulin, μIU/mL	2.9 (1.4, 6.7)	2.0 (0.7, 4.1)	3.6 (1.8, 8.0)	<0.001
Postprandial insulin, μIU/mL	3.9 (1.7, 15.0)	1.7 (0.7, 3.8)	8.1 (3.1, 21.1)	<0.001
MMTT stimulated C-peptide, pmol/L	70 (0, 350)	0 (0, 10)	25 (9, 540)	<0.001
Hypertension, n (%)	129 (29.9)	66 (34.2)	63 (26.5)	0.091
Diabetic retinopathy, n (%)	102 (30.6)	55 (39.9)	47 (24.1)	0.003
Carotid plaque, n (%)	213 (55.3)	110 (62.9)	103 (49.3)	0.010
Daily insulin dosage, U/d	33.0 (24.0, 42.0)	36.0 (28.3, 46.0)	29.4 (20.0, 40.0)	<0.001

Data are mean ± SD or median (IQR) unless otherwise indicated. IAA, insulin autoantibody; ICA, islet cell autoantibody; GAD autoantibody, glutamic acid decarboxylase autoantibody; SBP, systolic blood pressure; DBP, diastolic blood pressure; TG, triglyceride; LDL-C, low-density lipoprotein cholesterol; eGFR, estimated glomerular filtration rate; MMTT, mixed meal tolerance test.

After adjustment for age at diagnosis, duration of diabetes, BMI, GAD autoantibodies, eGFR, and HbA_1c_, three factors including duration of diabetes, GAD autoantibodies, and daily insulin dosage were still associated with lack of preserved C-peptide [OR 1.10 (IQR, 1.06–1.14); OR, 0.46 (IQR, 0.27–0.77); OR, 1.04 (IQR, 1.02–1.06)] **Table 2**.

**Table 2:** Variables independently associated with the preservation of C-peptide secretion.

	OR (95% CI)	*P*
Age at diagnosis	0.99 (0.97 to 1.01)	0.288
Duration of diabetes	1.10 (1.06 to 1.14)	<0.001
BMI	0.99 (0.92 to 1.07)	0.789
GAD autoantibody	0.46 (0.27 to 0.77)	0.003
eGFR	0.99 (0.98 to 1.00)	0.147
Daily insulin dosage	1.04 (1.02 to 1.06)	<0.001
HbA_1c_ > 7%	0.54 (0.26 to 1.14)	0.107

BMI, body mass index; GAD autoantibody, glutamic acid decarboxylase autoantibody; eGFR, estimated glomerular filtration rate.

### Sustained β-cell function associated with diabetes duration in the longitudinal cohort

The longitudinal analysis included 174 participants who had at least one follow-up visit with a fasting C-peptide test. The median follow-up was 2.0 years. **Supplementary Figure 3A** shown that β-cell function declined with increasing duration of diabetes. The proportions of participants with C-peptide >10 pmol/L were 71.4%, 56.8%, 71.7%, 62.5%, and 22.2% at baseline, < 1 year, 1 to 2 years, 2 to 3 years, 3 to 4 years, and 4 to 5 years follow-up, respectively (**Supplementary Figure 3B**). We divided these participants into two cohorts: those with failed β-cell function (ΔC-peptide _the last follow-up - baseline_ ≤ 0) and those with sustained β-cell function (ΔC-peptide _the last follow-up - baseline_ > 0). Cox regression analysis showed that duration of diabetes was independently associated with sustained β-cell function (**Supplementary Table 1**).

### Preserved C-peptide was associated with higher TIR

**Figure 2:**
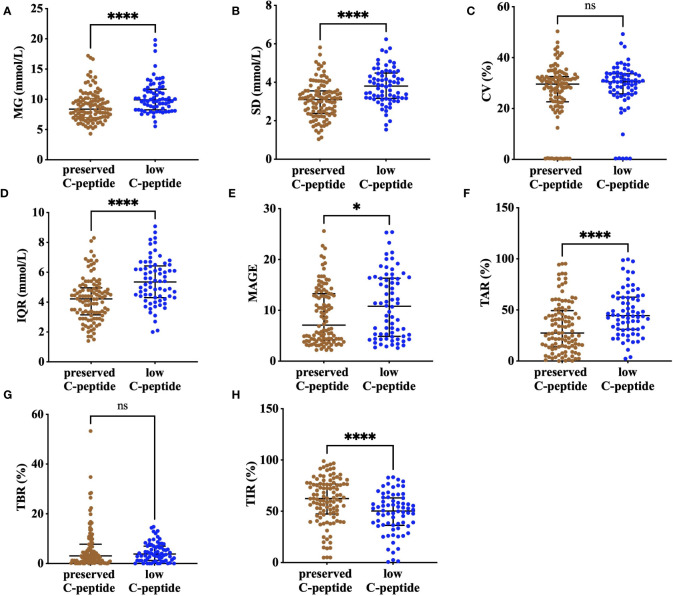
The comparison of CGM metrics, including MG **(A)**, SD **(B)**, CV **(C)**, IQR **(D)**, MAGE **(E)**, TAR **(F)**, TBR **(G)**, and TIR **(H)**, between the preserved–C-peptide group and the low–C-peptide group. **** means P<0.001, * means P<0.05, and ns means non-significant; MG, mean glucose; CV, coefficient of variance; IQR, interquartile range; SD, standard deviation; MAGE, mean amplitude of glycemia excursions; TBR, time below range (glucose concentrations below 3.9 mmol/L); TIR, time in range (glucose concentrations of 3.9–10.0 mmol/L); TAR, time above range (glucose concentrations over 10.0 mmol/L).

Mean glucose was lower in the preserved–C-peptide group compared with that in the low–C-peptide group [8.4 (IQR, 7.0–10.2) vs. 9.9 (IQR, 8.3–11.7) mmol/L, *P* <0.001]. In addition, TIR was higher, and TAR was lower in the preserved–C-peptide group [62.4 (IQR, 47.3–76.6) vs. 50.3 (IQR, 36.2–63.0) %, *P* <0.001; 27.4 (IQR, 14.0–49.3) vs. 44.4 (IQR, 31.0–62.5), *P* <0.001). Glucose metrics indicating variability, including SD, IQR, and MAGE, were lower in the preserved–C-peptide group [3.1 ± .9 vs.3.8 ± .9 mmol/L, *P* <0.001; 4.2 ± 1.4 vs. 5.4 ± 1.5 mmol/L, *P* < 0.001; 7.1 (IQR, 4.2–13.3) vs. 10.8 (IQR, 4.9–16.3), *P* = 0.029]. After adjustment of diabetes duration, preserved C-peptide was still associated with higher TIR and lower TAR, SD, and IQR (*P* =0.003, *P* =0.003, *P* = 0.03, *P* <0.001, and *P* <0.001) (**Figure 2**; **Supplementary Table 2**).

## Conclusions

Our study showed that preserved C-peptide secretion was common in Chinese individuals with T1D and was associated with diabetes duration, positive GAD autoantibody, and insulin dosage. Meanwhile, preserved C-peptide was also associated with favorable glycemic control as represented by TIR.

Persistent C-peptide secretion, reflecting some degree of intrinsic β-cell function, is now recognized to be common in T1D (5, 8, 10). In the Joslin Medalist Study, residual C-peptide secretion was detected in a large proportion of Medalists, even after more than 50 years of follow-up (9). However, such studies were mainly conducted in Caucasian populations, and few studies have focused on Chinese, with the currently available studies having relatively short diabetes duration or small populations. In a cohort of 446 participants with T1D with a mean duration of 2.36 years, more than 80% of them had detectable C-peptide, but the percentage decreased rapidly with disease progression (11). In another study of 109 participants with T1D followed for at least 10 years, Cheng et al. showed that 38.5% of participants had detectable C-peptide secretion (random C-peptide ≥16.7 pmol/L) (12). Miao and colleagues reported that, in 443 participants with T1D for 2.38 years, stimulated C-peptide ≥200 pmol/L was detected in 64.3% of participants (13). To our knowledge, our study was the largest with a relatively long duration of diabetes in the Chinese population with T1D and suggested that more than half of them still had preserved insulin secretion.

Previous studies have suggested that age at diagnosis, duration of diabetes, autoantibody positivity, and Human Leukocyte antigen (HLA) genotype may influence serum C-peptide level (10, 14, 15). Our results were consistent with the previous studies that diabetes duration was negatively associated with residual β-cell function and that autoantibody positivity was correlated with sustained intrinsic insulin production (1, 13, 16). The relationship between longer disease duration and lower C-peptide is widely recognized according to previous studies, whereas the finding of a strong relationship between higher autoantibody levels and higher C-peptide levels is difficult to interpret. Autoantibodies are generally good predictors of disease onset but are not specific for disease outcome (17, 18). Our previous findings showed that 17.1% of Chinese patients with T1D with long duration of diabetes were with GAD autoantibody positive, and 14.7% had fasted serum C-peptide higher than 75 pmol/L (19). Further investigation of GAD autoantibody is clearly required. Meanwhile, our study suggested that residual β-cell function was associated with lower daily insulin dose, which was in line with previous studies (11, 20–23). Because C-peptide levels represent intrinsic β-cell function (24), a possible explanation is that participants with higher C-peptide levels had more endogenous insulin production and required lower doses of exogenous insulin. As accumulating evidence suggests that preserved C-peptide is associated with a lower likelihood of diabetes microvascular complications (5, 10, 25), the association between autoantibody positivity, residual β-cell function, and favorable diabetes outcomes should be further investigated and the underlying mechanisms explored.

Understanding how the residual β-cell function relates to the heterogeneity of glycemic control is important for people with diabetes and their clinicians. Moreover, a more personalized approach to diabetes care may be possible with a better understanding of the contribution of residual β-cell function to CGM-derived metrics such as TBR, TIR, TAR, and CV. Previous studies have investigated the impact of residual insulin secretion in T1D, as measured by the MMTT, on the maintenance of glycemic control, as measured by HbA_1c_ (20, 26, 27). Previous studies in Caucasian populations have investigated the association between residual β-cell function and TIR. Researchers found that, in the T1D Exchange participants, fasting C-peptide was correlated with higher TIR (28). In addition, in a recent study recruiting participants from The Netherlands, Coco et al. suggested that residual insulin secretion, as measured by urinary C-peptide to creatinine ratio, was associated with longer TIR, shorter TBR and TAR, and lower CV (23). Although a study conducted in Chinese patients with diabetes including T1D, type 2 diabetes, and latent autoimmune diabetes in adults showed a continuous spectrum of glycemic variability pattern (29), no large-scale study focusing on T1D population in Chinese has been reported. Our study provided a relatively large sample size covering the entire duration of T1D in Chinese and showed that residual β-cell function was associated with TIR after adjustment for potential confounders.

This study had several limitations. First, the cross-sectional design made it impossible to establish causality. However, it is most likely that preserved β-cell function has a positive effect on glycemic control and not vice versa, as recent studies have shown that tight glycemic control, even with an artificial pancreas, does not preserve β-cell function even in newly diagnosed T1D subjects (30, 31). Second, although fasted serum may be a good representation of β-cell function, it is not considered the gold standard for measuring β-cell function. Therefore, we cannot exclude the possibility that our study underestimates the contribution of β-cell function to glycemic control. Third, our study was a single-center study, and the number of young patients with T1D was limited; we are planning on elaborate with some specialized children’s hospitals in the future study. Finally, as the CGM data were not blinded to the participants, other important confounders related to glycemic control, such as diabetes management skills, emotional factors could also contribute to the individual’s CGM metrics. However, as we used professional CGM in the study and all participants received standard T1D care from our specialists, the impact of individual procedures was minimized. Nevertheless, we point out that this observation further supports the concept that β-cell function contributes to better daily control, as we found strong and consistent associations with both TIR and TAR.

In conclusion, residual β-cell function was common in people with T1D, and preservation of C-peptide secretion was associated with shorter duration, positive GAD autoantibody, and lower insulin dosage. As glucose control measured by CGM is at least partly influenced by residual β-cell function, personalized glucose targets should be considered on the basis of individual C-peptide level. Furthermore, disease-modifying therapies aiming to preserve β-cell function should also be considered in the future.

## Data availability statement

The raw data supporting the conclusions of this article will be made available by the authors, without undue reservation.

## Ethics statement

The studies involving humans were approved by Peking University People’s Hospital Ethics Committee. The studies were conducted in accordance with the local legislation and institutional requirements. The human samples used in this study were acquired from a by-product of routine care or industry. Written informed consent for participation was not required from the participants or the participants’ legal guardians/next of kin in accordance with the national legislation and institutional requirements.

## Author contributions

WL: Conceptualization, Methodology, Writing – original draft, Writing – review & editing. YF: Data curation, Formal analysis, Visualization, Writing – original draft. XC: Conceptualization, Methodology, Writing – review & editing. YZ: Investigation, Writing – review & editing. MZ: Investigation, Writing – review & editing. XH: Investigation, Writing – review & editing. JL: Investigation, Writing – review & editing. SY: Investigation, Writing – review & editing. DC: Software, Writing – review & editing. JC: Software, Writing – review & editing. LW: Software, Writing – review & editing. DS: Software, Writing – review & editing. LJ: Conceptualization, Methodology, Supervision, Writing – review & editing.
