# Supplementary figures and images for: Preserved C-peptide is common and associated with higher time in range in Chinese type 1 diabetes
Source: Front Endocrinol (Lausanne). 2024 Feb 9;15:1335913. doi: 10.3389/fendo.2024.1335913 (PMC10884320; doi:10.3389/fendo.2024.1335913)

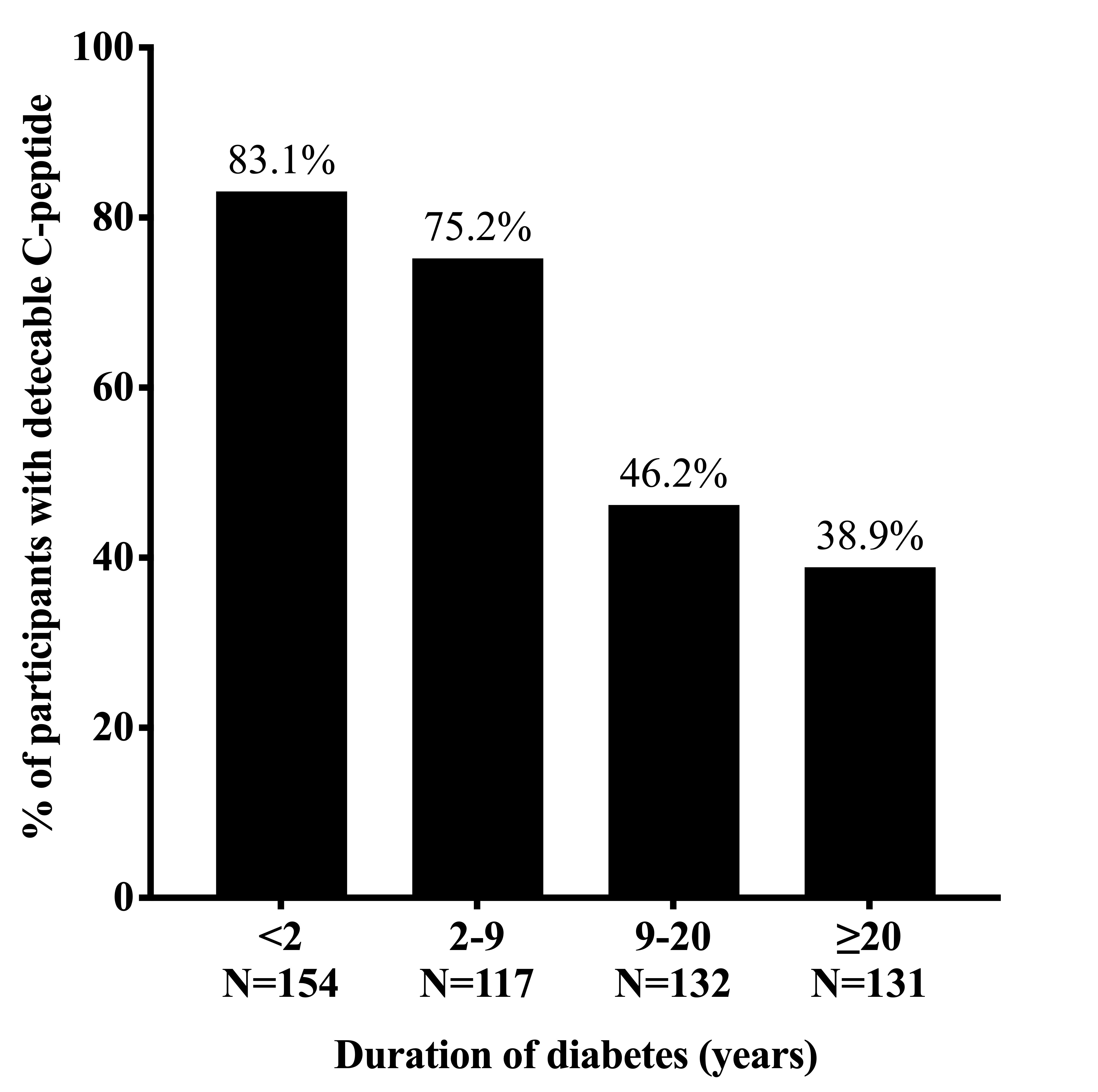

Supplement: Supplementary file 1 [file Image_1.jpeg]

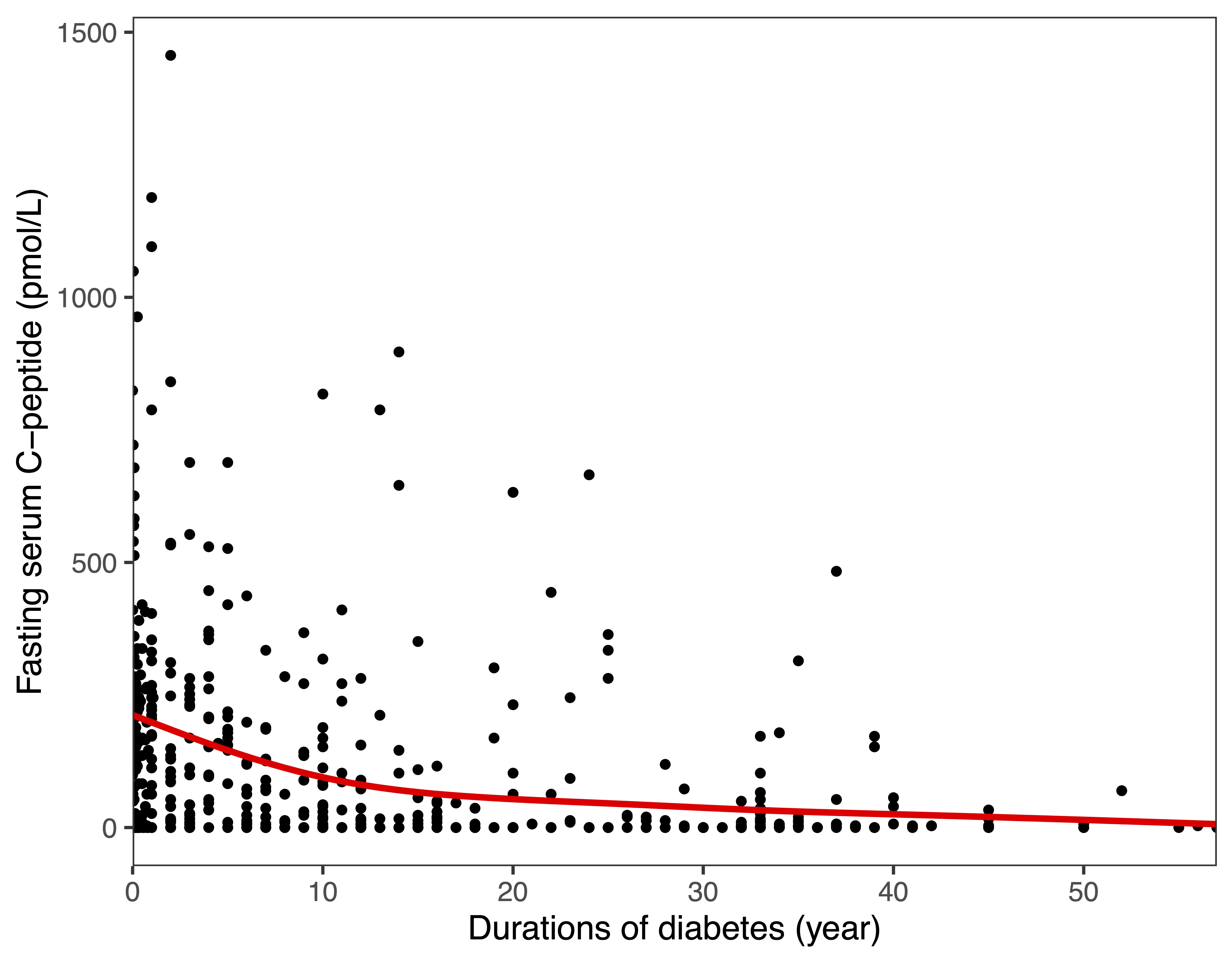

Supplement: Supplementary file 2 [file Image_2.jpeg]

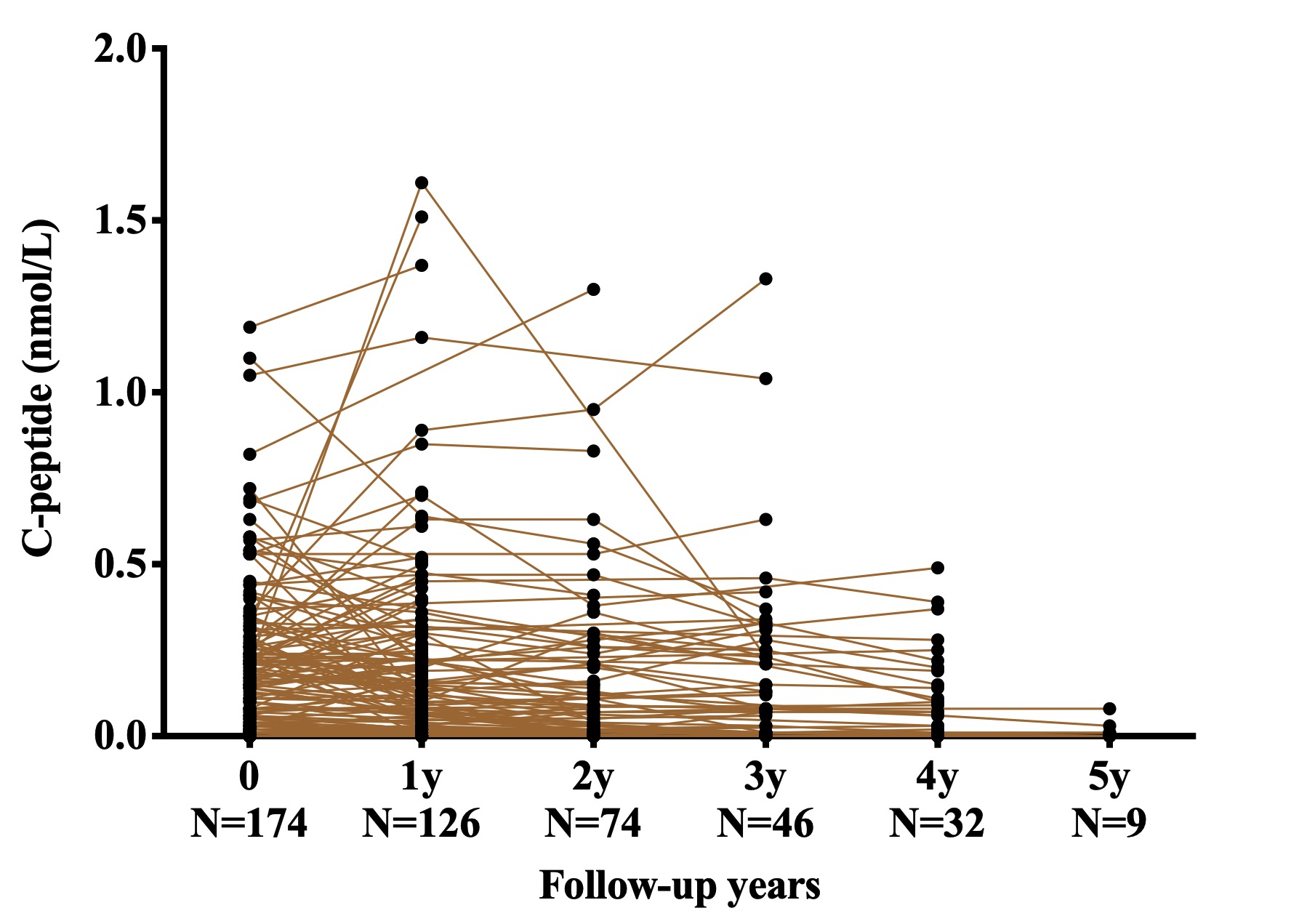

Supplement: Supplementary file 3 [file Image_3.jpeg]

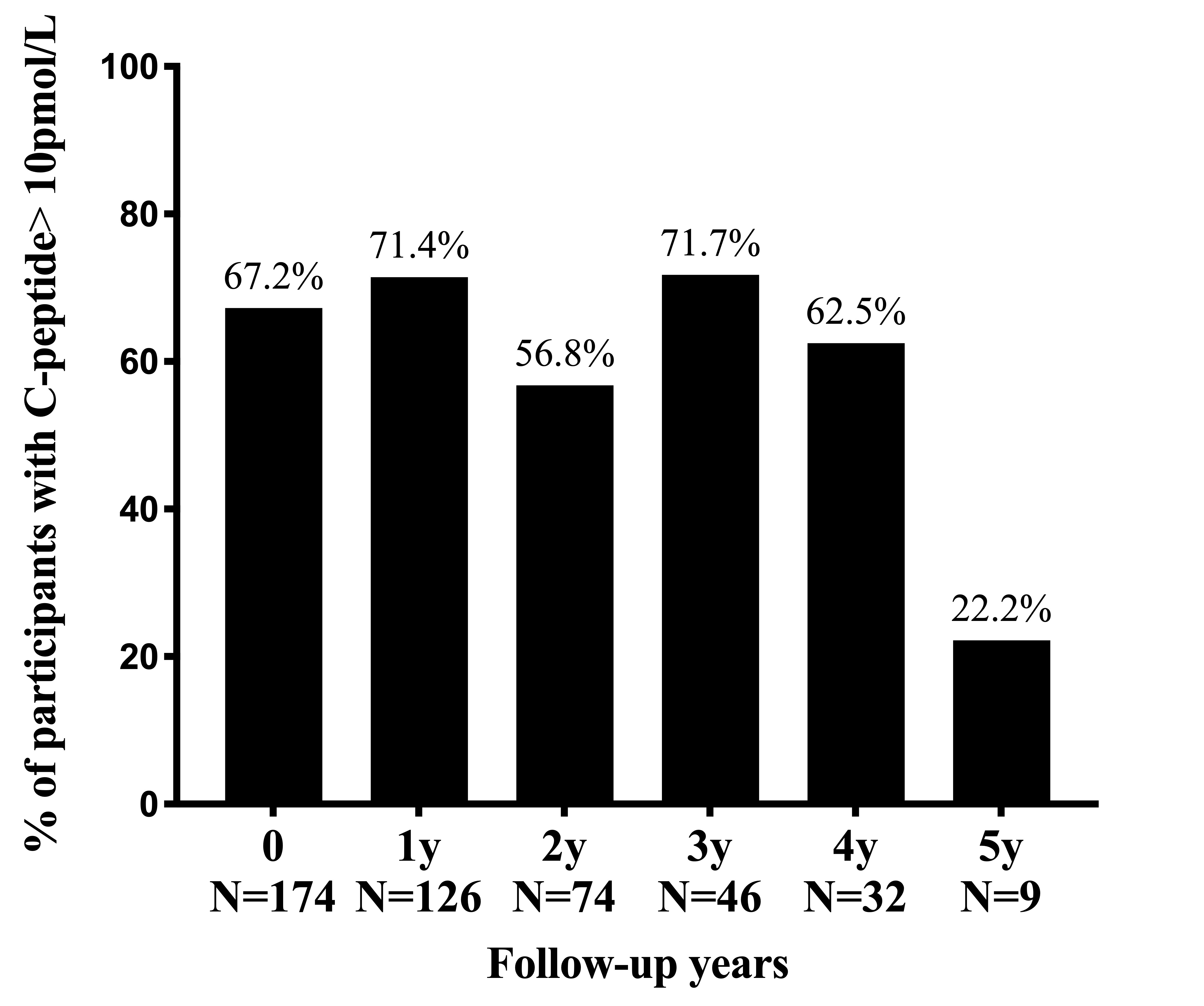

Supplement: Supplementary file 4 [file Image_4.jpeg]
