# Supplementary material for: Preserved C-peptide is common and associated with higher time in range in Chinese type 1 diabetes
Source: Front Endocrinol (Lausanne). 2024 Feb 9;15:1335913. doi: 10.3389/fendo.2024.1335913 (PMC10884320; doi:10.3389/fendo.2024.1335913)
Supplement: Supplementary file 5 [file Table_1.docx]

| Supplementary Table 1- Cox regression analysis of the loss of C-peptide in patients with type 1 diabetes | | | | | |
| --- | --- | --- | --- | --- | --- |
|  | Total  N=174 | Failed β function  N=129 | Sustained β function  N=45 | OR (95% CI) | *P* |
| Age at diagnosis, n (%) |  |  |  |  |  |
| 7 y | 1 (0.6) | 1 (0.8) | 0 (0) | 1 | 0.775 |
| 7-12 y | 7 (4.0) | 5 (3.9) | 2 (4.4) | 0.463 (0.051 to 4.230) | 0.495 |
| 13 y | 166 (95.4) | 123 (95.3) | 43 (95.6) | 0.484 (0.063 to 3.697) | 0.484 |
| Duration of diabetes, n (%) |  |  |  |  |  |
| 1 y | 45 (25.9) | 35 (27.1) | 10 (22.2) | 1 | 0.001 |
| 1-5 y | 53 (30.5) | 38 (29.5) | 15 (33.3) | 0.380 (0.227 to 0.636) | 0.001 |
| 5-10 y | 26 (14.9) | 17 (13.2) | 9 (20.0) | 0.384 (0.205 to 0.721) | 0.003 |
| 10 y | 50 (28.7) | 39 (30.2) | 11 (24.4) | 0.415 (0.246 to 0.700) | 0.001 |
| HbA_1c_$>$7%, n (%) | 151 (87.3) | 113 (88.3) | 38 (84.4) | 0.919 (0.502 to 1.684) | 0.786 |
| GAD autoantibody positivity, n (%) | 116 (72.0) | 86 (72.9) | 30 (69.8) | 0.837 (0.540 to 1.298) | 0.427 |
| The impact of age at diagnosis, duration of diabetes, HbA1c and GADA on β-cell function was analyzed using a cox regression model. GAD autoantibody: glutamic acid decarboxylase autoantibody. | | | | | |
