# Supplementary material for: Preserved C-peptide is common and associated with higher time in range in Chinese type 1 diabetes
Source: Front Endocrinol (Lausanne). 2024 Feb 9;15:1335913. doi: 10.3389/fendo.2024.1335913 (PMC10884320; doi:10.3389/fendo.2024.1335913)
Supplement: Supplementary file 6 [file Table_2.docx]

| Supplementary Table 2- Comparison of data derived from isCGM by C-peptide category | | | | | |
| --- | --- | --- | --- | --- | --- |
|  | Total  N=178 | Low C-peptide  10 pmol/L  N=70 | Preserved C-peptide  10 pmol/L  N=108 | *P* | Adjust *P* |
| MG (mmol/l) | 9.1 (7.6, 10.5) | 9.9 (8.3, 11.7) | 8.4 (7.0, 10.2) | 0.001 | 0.001 |
| SD (mmol/l) | 3.3 (1.0) | 3.8 (0.9) | 3.1 (0.9) | 0.001 | 0.001 |
| CV (%) | 30.4 (24.5, 33.7) | 30.5 (25.7, 33.7) | 29.6 (22.7, 32.6) | 0.244 | 0.179 |
| TBR (%) | 3.5 (1.2, 7.8) | 3.8 (1.2, 7.0) | 3.1 (0.9, 7.8) | 0.911 | 0.311 |
| TIR (%) | 57.5 (44.1, 73.3) | 50.3 (36.2, 63.0) | 62.4 (47.3, 76.6) | 0.001 | 0.003 |
| TAR (%) | 35.9 (18.2, 52.4) | 44.4 (31.0, 62.5) | 27.4 (14.0, 49.3) | 0.001 | 0.003 |
| IQR (mmol/l) | 4.6 (1.6) | 5.4 (1.5) | 4.2 (1.4) | 0.001 | 0.001 |
| MAGE | 8.1 (4.3, 14.6) | 10.8 (4.9, 16.3) | 7.1 (4.2, 13.3) | 0.029 | 0.056 |
| Analysis of association between residual β-cell function by categories of fasting C-peptide and MG, SD, CV, TBR, TIR, TAR, IQR, and MAGE. Fasting serum C-peptide values are defined as low ($\leq$10 pmol/L) and preserved ($>$10 pmol/L). MG, mean glucose; TBR, time below range (glucose concentrations below 3.9 mmol/L); TIR, time in range (glucose concentrations of 3.9-10.0 mmol/L); TAR, time above range (glucose concentrations over 10.0 mmol/L); SD, standard deviation; CV, coefficient of variation; IQR, interquartile range; MAGE, mean amplitude of glycemic excursion. *P* value were adjusted by duration of diabetes. | | | | | |
